# Type 2 diabetes, depressive symptoms and trajectories of cognitive decline in a national sample of community-dwellers: A prospective cohort study

**DOI:** 10.1371/journal.pone.0175827

**Published:** 2017-04-17

**Authors:** Panayotes Demakakos, Graciela Muniz-Terrera, Arie Nouwen

**Affiliations:** 1 Department of Epidemiology and Public Health, University College London, London, United Kingdom; 2 Centre for Dementia Prevention, University of Edinburgh, Edinburgh, United Kingdom; 3 Department of Psychology, Middlesex University, London, United Kingdom; Nathan S Kline Institute, UNITED STATES

## Abstract

We examined the individual and synergistic effects of type 2 diabetes and elevated depressive symptoms on memory and executive function trajectories over 10 and eight years of follow-up, respectively. Our sample comprised 10,524 community-dwellers aged ≥50 years in 2002–03 from the English Longitudinal Study of Ageing. With respect to memory (word recall), participants with either diabetes or elevated depressive symptoms recalled significantly fewer words compared with those free of these conditions (reference category), but more words compared with those with both conditions. There was a significant acceleration in the rate of memory decline in participants aged 50–64 years with both conditions (-0.27, 95% CI, -0.45 to -0.08, per study wave), which was not observed in those with either condition or aged ≥65 years. With respect to executive function (animal naming), participants aged ≥65 years with diabetes or those with elevated depressive symptoms named significantly fewer animals compared with the reference category, while those with both conditions named fewer animals compared with any other category. The rate of executive function decline was significantly greater in participants with both conditions (-0.54, 95% CI, -0.99 to -0.10; and –0.71, 95% CI, -1.16 to -0.27, per study wave, for those aged 50–64 and ≥65 years, respectively), but not in participants with either condition. Diabetes and elevated depressive symptoms are inversely associated with memory and executive function, but, individually, do not accelerate cognitive decline. The co-occurrence of diabetes and elevated depressive symptoms significantly accelerates cognitive decline over time, especially among those aged 50–64 years.

## Introduction

Depression is almost twice as common in type 2 diabetes mellitus (hereafter diabetes) patients compared with people free of diabetes [1]. The relationship between diabetes and depression appears to be bidirectional [2,3] with depression increasing the risk of incident diabetes [3,4] and diabetes increasing the risk of subsequent depression [5,6]. Recent findings expands on this evidence and suggest that the bidirectional association is age dependent with diagnosed diabetes being associated with subsequent elevated depressive symptoms in people aged 52 to 64 years, but not those aged ≥65 years [7]. Both diabetes and depression are associated with increased risk of cognitive decline and dementia [8–16].

Despite the close relationship between diabetes and depression and their importance for cognition, few prospective studies have examined the combined effect of diabetes and depression on cognitive decline and dementia. Two studies found that comorbid depression was associated with a 2- to 3-fold increased risk of developing dementia in people with diabetes [17,18], while a large-scale study that used data from national registries found the combined effect of diabetes and depression on dementia risk to be greater than the sum of their individual effects [19]. With respect to cognitive decline, a study reported that diabetes patients with comorbid depression experienced a greater cognitive decline compared with those without depression over 40-month follow-up [20]. A recent study of community-dwelling Mexican Americans aged ≥65 years found that comorbid depression and diabetes were associated with worse cognitive performance over an 11-year follow-up [21].

We used a national sample of community-dwelling individuals aged ≥50 years to investigate the associations between baseline diabetes and elevated depressive symptoms and trajectories of cognitive function over a 10-year follow-up. We examined differences in the risk of cognitive decline as well as the rate of cognitive decline according to baseline diabetes and elevated depressive symptoms with people with both conditions constituting a separate category.

## Research design and methods

### Study population

The sample comes from the English Longitudinal Study of Ageing (ELSA), a prospective observational study of community-dwelling people aged ≥50 years in England, and was recruited using a multistage stratified random probability design. The baseline interview took place in 2002–03 and sample comprised 11,391 individuals. After the baseline, follow-up interviews took place biennially. All waves and parts of ELSA have been approved by the national Research Ethics Service (http://www.hra.nhs.uk/about-the-hra/our-committees/res/) and informed consent has been obtained by the participants after they were informed in writing and in advance about the study and its purpose. Written informed consent was sought for specific parts of the study including the health examination; for the interview the informed consent was verbal.

A detailed description of the study can be found at: http://www.elsa-project.ac.uk/. The analytical sample comprised 10,524 participants who were present at baseline, after the exclusion of 362 participants with proxy or partial interviews, 63 possible cases of type 1 diabetes i.e. age at self-reported diabetes diagnosis ≤40 years, 44 cases of self-reported dementia and Alzheimer’s disease, and 398 participants with missing values in baseline variables (excluding BMI).

### Assessment of cognitive function

We assessed two domains of cognitive function: memory and executive function. We measured two dimensions of memory, immediate and delayed recall, using a 10-word test that has earlier been used in the Health and Retirement Study (HRS) [22]. Participants were asked to listen to a list of 10 words and immediately recall as many as they could. Few minutes later, without prior notice and after they had engaged in other tasks, they were asked again to recall as many words as they could. Because the associations between diabetes and elevated depressive symptoms and immediate and delayed recall scores followed similar patterns and our objective was to examine the overall effect of comorbid depression and diabetes on memory decline, we combined the two recall scores to generate a recall summary score (range: 0 to 20 words), which we then used in our analyses. We assessed executive function using a semantic verbal fluency test, which involved naming as many animals as one could in 60 seconds [22]. At baseline, the animal naming score ranged from 0 to 50 words. Word recall ability was measured at baseline and all five follow-up interviews, and animal naming at baseline and the first four follow-up interviews. To minimise non-response bias, we imputed the missing values in both cognitive scores using chained equations in STATA 14. The imputed data were censored at the date of death. The analyses were based on 57,199 observations of recall score of which 14,652 were imputed and 48,791 observations of animal naming score of which 11,375 were imputed.

### Assessment of diabetes and elevated depressive symptoms

We measured depressive symptoms at baseline using the eight-item Center for Epidemiological Studies-Depression (CES-D) scale [23]. We derived a CES-D summary score by adding up responses to all eight dichotomous questions. To generate a variable of elevated depressive symptoms, we dichotomized the CES-D summary score using the cut point of ≥4, which corresponds to the cut point of ≥16 on the 20-item CES-D [23]. We also measured self-reported doctor-diagnosed diabetes at baseline. We were able to validate baseline diabetes diagnosis for 8,081 of our participants using diabetes medication data that predated the baseline (they were measured in 1998 or 2001). We combined information on diabetes and elevated depressive symptoms to derive the main exposure variable with the following categories: 1) without diabetes and elevated depressive symptoms, 2) without elevated depressive symptoms, but with diabetes, 3) with elevated depressive symptoms, but without diabetes, and 4) with both diabetes and elevated depressive symptoms.

### Covariates

Age, sex, marital status, education, and occupational class, self-reported doctor-diagnosed chronic conditions (i.e. heart disease, stroke, hypertension, and chronic lung disease), smoking, physical activity, alcohol consumption, and measured body mass index (BMI) were used in the analyses. All covariates were measured at baseline in 2002–03, except for BMI, which calculation was based on height and weight measurements that were taken by nurses in 1998, 1999 or 2001. To avoid unnecessarily excluding from our analyses a large number of participants with missing BMI values (n = 1062), we imputed missing BMI values.

### Statistical analysis

We analysed the baseline characteristics of the sample according to diabetes and elevated depressive symptoms status. We estimated a linear mixed (random coefficient) model of the association between the exposure and six repeated measurements of word recall over a 10-year follow-up that was gradually adjusted for covariates. Time was measured using a variable that reflected the chronological order of the six repeated measurements (study waves) i.e. *t =* 1, 2, 3, 4, 5, 6. To explore whether baseline diabetes and elevated depressive symptoms accelerated memory decline over time, we also estimated a model that included the interaction term exposure*time. The same modelling approach was employed when analysing the association between the exposure and five repeated measurements of animal naming score over eight years of follow-up. Because the association between diabetes and elevated depressive symptoms varied by age in our data [7] and we found that the association between the predictor and memory, but not executive function, varied by age, we stratified our analyses by age into two groups: 50–64 and ≥65 years. The use of 65 years as a cut point was decided on an empirical basis i.e. was the mean age of the sample, used to be the national pension age for men, and resulted in two groups of similar size. For comparison reasons, we performed additional analyses where we exclusively used the observed data (see Tables A-D in S1 File).

## Results

Participants with both diabetes and elevated depressive symptoms were more likely to be of lower socioeconomic status, physically inactive, and obese and less likely to consume alcohol and be married. Participants with diabetes, but without elevated depressive symptoms, were more likely to be older, male and have hypertension, while those with elevated depressive symptoms, but without diabetes, were more likely to be female and current smokers (Table 1).

**Table 1:** The baseline characteristics of 10524 women and men aged ≥50 years by type 2 diabetes and elevated depressive symptoms.

	Without diabetes and elevated depressive symptoms (n = 8275)	With diabetes, but without elevated depressive (n = 554)	Without diabetes, but with elevated depressive symptoms (n = 1526)	With both diabetes and elevated depressive symptoms (n = 169)	P value^a^
**Mean age, years, (SD)**	64.4 (9.9)	68.4 (9.1)	65.9 (10.8)	67.4 (9.3)	<0.001
**Sex (%)**					<0.001
Male	3910 (47.3)	340 (61.4)	526 (34.5)	71 (42.0)	
Female	4365 (52.7)	214 (38.6)	1000 (65.5)	98 (58.0)	
**Marital status (%)**					<0.001
Married	5784 (69.9)	398 (71.8)	779 (51.0)	78 (46.1)	
Other	2491 (30.1)	156 (28.2)	747 (49.0)	91 (53.9)	
**Education (%)**					<0.001
A-level or higher	2562 (31.0)	135 (24.4)	281 (18.4)	25 (14.8)	
Secondary or equivalent	2563 (31.0)	156 (28.1)	377 (24.7)	34 (20.1)	
No qualifications	3150 (38.0)	263 (47.5)	868 (56.9)	110 (65.1)	
**Occupational class (%)**					<0.001
Managerial and professional occupations	2644 (32.0)	164 (29.6)	304 (19.9)	26 (15.4)	
Intermediate occupations	2046 (24.7)	107 (19.3)	330 (21.6)	20 (11.8)	
Semi-routine and routine occupations	3585 (43.3)	283 (50.1)	892 (58.5)	123 (72.8)	
**Physical activity at least once a week (%)**					<0.001
Vigorous-intensity	2583 (31.2)	96 (17.3)	236 (15.5)	11 (6.5)	
Moderate-intensity	4054 (49.0)	277 (50.0)	637 (41.7)	58 (34.3)	
Mild-intensity	1020 (12.3)	108 (19.5)	356 (23.3)	50 (29.6)	
Physically inactive	618 (7.5)	73 (13.2)	297 (19.5)	50 (29.6)	
**Smoking (%)**					<0.001
Current smoker	1378 (16.7)	80 (14.4)	408 (26.7)	33 (19.5)	
Former smoker	3823 (46.2)	323 (58.3)	680 (44.6)	83 (49.1)	
Never smoker	3074 (37.1)	151 (27.3)	438 (28.7)	53 (31.4)	
**Body mass index**^b^ **(%)**					<0.001
<25kg/m^2^	2311 (27.9)	76 (13.7)	406 (26.6)	18 (10.7)	
25 to <30 kg/m^2^	3470 (41.9)	216 (39.0)	540 (35.4)	45 (26.6)	
≥ 30 kg/m2	1736 (21.0)	201 (36.3)	370 (24.2)	73 (43.2)	
Missing	758 (9.2)	61 (11.0)	210 (13.8)	33 (19.5)	
**Frequency of alcohol consumption (%)**					<0.001
Daily or almost daily	2491 (30.1)	115 (20.8)	346 (22.7)	18 (10.6)	
1–2 times a week or monthly	3533 (42.7)	221 (39.9)	526 (34.5)	51 (30.2)	
Never or almost never	2251 (27.2)	218 (39.3)	654 (42.8)	100 (59.2)	
**Heart disease (%)**					<0.001
No	7856 (94.9)	464 (83.8)	1384 (90.7)	145 (85.8)	
Yes	419 (5.1)	90 (16.2)	142 (9.3)	24 (14.2)	
**Stroke (%)**					<0.001
No	8002 (96.7)	517 (93.3)	1428 (93.6)	147 (87.0)	
Yes	273 (3.3)	37 (6.7)	98 (6.4)	22 (13.0)	
**Hypertension (%)**					<0.001
No	5414 (65.4)	196 (35.4)	881 (57.7)	66 (39.1)	
Yes	2861 (34.6)	358 (64.6)	645 (42.3)	103 (60.9)	
**Chronic Lung Disease (%)**					<0.001
No	7830 (94.6)	512 (92.4)	1342 (87.9)	148 (87.6)	
Yes	445 (5.4)	42 (7.6)	184 (12.1)	21 (12.4)	

^a^ P values were calculated using chi-square, Kruskal-Wallis and analysis of variance tests for categorical, ordinal and continuous covariates, respectively.

^b^ The missing category was not used in the calculation of the P value

**Fig 1:**
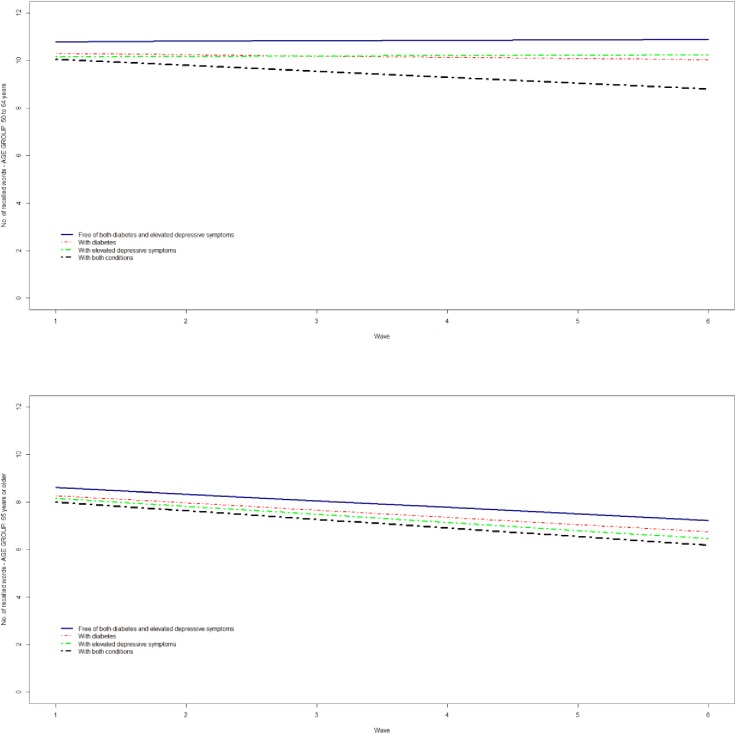
The trajectories of memory (word recall summary score) by diabetes and elevated depressive symptoms among participants aged 50 to 64 (top panel) and 65 years or older (bottom panel).

**Fig 2:**
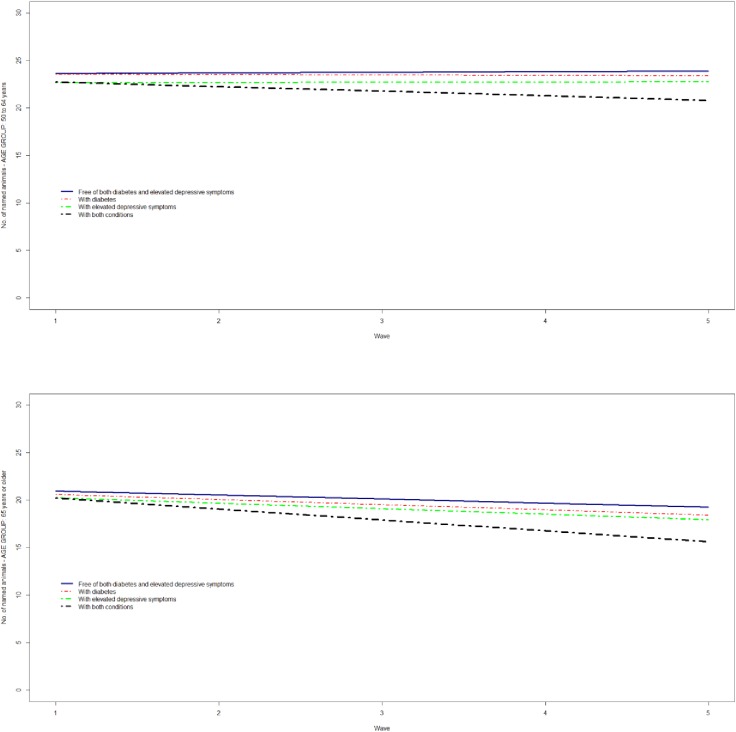
The trajectories of executive function (animal naming score) by diabetes and elevated depressive symptoms among participants aged 50 to 64 (top panel) and 65 years or older (bottom panel).

The memory and executive function of participants with both diabetes and elevated depressive symptoms at baseline significantly deteriorated over time compared with the reference category (Figs 1 and 2), except for memory among those aged ≥65 years.

In terms of memory, participants aged 50–64 years with both conditions recalled on average 1.25 fewer words (95% CI, -1.83 to -0.68) compared with the reference category after adjustment for covariates. The respective estimate for participants aged ≥65 years was 0.74 fewer words (95% CI, -1.28 to -0.20) (Table 2). In both age groups, participants with either condition did better than those with both conditions, but significantly worse compared with the reference category. There was a significant acceleration in the rate of memory decline in participants aged 50–64 years with both diabetes and elevated depressive symptoms (-0.27; 95% CI, -0.45 to -0.08, per study wave) compared with the reference category, which was not observed in those with either condition or aged ≥65 years (Table 2).

**Table 2:** The prospective association between type 2 diabetes, elevated depressive symptoms and word recall summary score (memory) over 10 years in 10524 participants aged ≥50 years.

	Without diabetes and elevated depressive symptoms	With diabetes, but without elevated depressive	Without diabetes, but with elevated depressive symptoms	With both diabetes and elevated depressive symptoms
	**Participants aged 50 to 64 (n = 5512)**			
**No. of participants**	4493	187	761	71
**Slope (rate of decline)**^a^				
**Model 1**^b^	1.00 (reference)	-0.96 (-1.32 to -0.60)***	-1.19 (-1.39 to -0.99)***	-2.14 (-2.76 to -1.52)***
**Model 2**^c^	1.00 (reference)	-0.84 (-1.20 to -0.47)***	-1.11 (-1.32 to -0.91)***	-2.04 (-2.66 to -1.42)***
**Model 3**^d^	1.00 (reference)	-0.76 (-1.09 to -0.42)***	-0.77 (-0.96 to -0.58)***	-1.52 (-2.10 to -0.94)***
**Model 4**^e^	1.00 (reference)	-0.65 (-0.98 to -0.31)***	-0.64 (-0.83 to -0.45)***	-1.25 (-1.83 to -0.68)***
**Model 5**^f^	1.00 (reference)	-0.44 (-0.87 to 0.0001)*	-0.63 (-0.88 to -0.39)***	-0.48 (-1.21 to 0.25)
Slope acceleration (exposure*time interaction)^a^				
**Model 5**^f^	1.00 (reference)	-0.07 (-0.17 to 0.03)	-0.002 (-0.06 to 0.06)	-0.27 (-0.45 to -0.08)**
	**Participants aged ≥65 years (n = 5012)**			
**No. of participants**	3782	367	765	98
**Slope (rate of decline)**^a^				
**Model 1**^b^	1.00 (reference)	-0.64 (-0.94 to -0.34)***	-1.03 (-1.25 to -0.81)***	-1.57 (-2.13 to -1.00)***
**Model 2**^c^	1.00 (reference)	-0.61 (-0.92 to -0.31)***	-1.01 (-1.22 to -0.79)***	-1.46 (-2.03 to -0.89)***
**Model 3**^d^	1.00 (reference)	-0.49 (-0.77 to -0.20)***	-0.71 (-0.91 to -0.50)***	-1.01 (-1.56 to -0.47)***
**Model 4**^e^	1.00 (reference)	-0.39 (-0.67 to -0.10)**	-0.55 (-0.76 to -0.33)***	-0.74 (-1.28 to -0.20)**
**Model 5**^f^	1.00 (reference)	-0.32 (-0.69 to 0.05)	-0.39 (-0.66 to -0.13)**	-0.53 (-1.20 to 0.15)
Slope acceleration (exposure*time interaction)^a^				
**Model 5**^f^	1.00 (reference)	-0.03 (-0.14 to 0.09)	-0.06 (-0.13 to 0.01)	-0.09 (-0.27 to 0.10)

^a^ The estimates are β regression coefficient (95% confidence intervals)

^b^ Model 1 is adjusted for age, sex and marital status

^c^ Model 2 is adjusted for age, sex, marital status and self-reported chronic conditions i.e. heart disease, stroke, hypertension and chronic lung disease

^d^ Model 3 is adjusted for age, sex, marital status, self-reported chronic conditions i.e. heart disease, stroke, hypertension and chronic lung disease, education and occupational class

^e^ Model 4 is adjusted for age, sex, marital status, self-reported chronic conditions i.e. heart disease, stroke, hypertension and chronic lung disease, education, occupational class, physical activity, smoking, alcohol consumption, and body mass index

^f^ Model 5 is adjusted for all covariates included in Model 4 plus adjustment for the exposure*time interaction term

*** p value≤0.001

** p value≤0.01

* p value≤0.05

With respect to executive function, participants aged 50–64 years with both conditions on average named 1.73 fewer animals (95% CI, -2.94 to -0.51) compared with the reference category after adjustment for covariates, while those aged ≥65 years with both conditions named 1.60 fewer animals (95% CI, -2.57 to -0.63) (Table 3). Participants with elevated depressive symptoms, and those aged ≥65 years with diabetes, also named significantly fewer animals compared with the reference category after adjustment for covariates. In both age groups, the rate of executive function decline was significantly greater in participants with both conditions compared with the reference category (-0.54; 95% CI, -0.99 to -0.10; and -0.71; 95% CI, -1.16 to -0.27, per study wave, for those aged 50–64 and ≥65 years, respectively), but not those with either diabetes or elevated depressive symptoms (Table 3).

**Table 3:** The prospective association between type 2 diabetes, elevated depressive symptoms and animal naming score (executive function) over 8 years in 10524 participants aged ≥50 years.

	Without diabetes and elevated depressive symptoms	With diabetes, but without elevated depressive	Without diabetes, but with elevated depressive symptoms	With both diabetes and elevated depressive symptoms
	**Participants aged 50 to 64 (n = 5512)**			
**No. of participants**	4493	187	761	71
**Slope (rate of decline)**^a^				
**Model 1**^b^	1.00 (reference)	-0.65 (-1.43 to 0.13)	-2.01 (-2.43 to -1.59)***	-3.22 (-4.49 to -1.95)***
**Model 2**^c^	1.00 (reference)	-0.49 (-1.28 to 0.30)	-1.89 (-2.31 to -1.47)***	-3.07 (-4.35 to -1.79)***
**Model 3**^d^	1.00 (reference)	-0.34 (-1.08 to 0.41)	-1.27 (-1.67 to -0.87)***	-2.13 (-3.34 to -0.92)***
**Model 4**^e^	1.00 (reference)	-0.25 (-0.99 to 0.50)	-1.04 (-1.45 to -0.63)***	-1.73 (-2.94 to -0.51)**
**Model 5**^f^	1.00 (reference)	-0.01 (-0.96 to 0.95)	-0.95 (-1.45 to -0.44)***	-0.38 (-1.92 to 1.17)
Slope acceleration (exposure*time interaction)^a^				
**Model 5**^**f**^	1.00 (reference)	-0.09 (-0.36 to 0.17)	-0.04 (-0.18 to 0.10)	-0.54 (-0.99 to -0.10)^*^
	**Participants aged ≥65 years (n = 5012)**			
**No. of participants**	3782	367	765	98
**Slope (rate of decline)**^a^				
**Model 1**^b^	1.00 (reference)	-0.94 (-1.47 to -0.41)***	-1.72 (-2.13 to -1.32)***	-2.97 (-3.99 to -1.95)***
**Model 2**^c^	1.00 (reference)	-0.88 (-1.41 to -0.34)***	-1.66 (-2.07 to -1.26)***	-2.73 (-3.75 to -1.71)***
**Model 3**^d^	1.00 (reference)	-0.69 (-1.20 to -0.18)**	-1.22 (-1.61 to -0.82)***	-2.10 (-3.07 to -1.12)***
**Model 4**^e^	1.00 (reference)	-0.53 (-1.04 to -0.02)*	-0.91 (-1.31 to -0.52)***	-1.60 (-2.57 to -0.63)***
**Model 5**^f^	1.00 (reference)	-0.28 (-0.95 to 0.40)	-0.59 (-1.08 to -0.09)*	-0.06 (-1.36 to 1.24)
Slope acceleration (exposure*time interaction)^a^				
**Model 5**^f^	1.00 (reference)	-0.12 (-0.34 to 0.11)	-0.15 (-0.32 to 0.03)	-0.71 (-1.16 to -0.27)**

^a^ The estimates are β regression coefficient (95% confidence intervals)

^b^ Model 1 is adjusted for age, sex and marital status

^c^ Model 2 is adjusted for age, sex, marital status and self-reported chronic conditions i.e. heart disease, stroke, hypertension and chronic lung disease

^d^ Model 3 is adjusted for age, sex, marital status, self-reported chronic conditions i.e. heart disease, stroke, hypertension and chronic lung disease, education and occupational class

^e^ Model 4 is adjusted for age, sex, marital status, self-reported chronic conditions i.e. heart disease, stroke, hypertension and chronic lung disease, education, occupational class, physical activity, smoking, alcohol consumption, and body mass index

^f^ Model 5 is adjusted for all covariates included in Model 4 plus adjustment for the exposure*time interaction term

*** p value≤0.001

** p value≤0.01

* p value≤0.05

## Discussion

In a national sample of community-dwelling people aged ≥50 years we found that the co-occurrence of diabetes and elevated depressive symptoms exerts a synergistic effect on cognitive ability and accelerates cognitive decline over time. Participants with either diabetes or elevated depressive symptoms also performed worse on both tests compared with participants who were free of these conditions, but their cognitive ability did not decline any faster.

To our knowledge our study is the first to examine the synergistic effect of diabetes and elevated depressive symptoms on cognitive decline in a national sample of community-dwelling people aged ≥50 years. Our findings concur with findings suggesting that the combination of diabetes and depression is prospectively associated with greater cognitive decline in diabetes patients [20] and an increased risk of dementia in diabetes patients [17,18] and the general population [19], but are at odds with those of a study of 570 diabetes patients and controls that did not find any difference in the cognitive performance of diabetes patients according to elevated depressive symptoms [24]. They also partially concur with the findings of a recent study of 2,756 community-dwelling Mexican Americans aged ≥65 years [21]. Like our study, this study found that the co-existence of diabetes and depression is associated with greater cognitive decline over time, but also that diabetes is associated with greater cognitive decline independent of comorbid depression, which is something that we did not find in our data. We hypothesise that the discrepancy between these findings and our findings is mostly related to differences in the epidemiological profiles and other characteristics of the two samples as well as the use of different outcome measures.

In our study, elevated depressive symptoms were inversely associated with both memory and executive function. Although research tends to differentiate between early- and late-onset depression as predictors of cognitive decline and dementia and the role of late-life depression in cognitive decline and dementia remains unclear [15,16], in agreement with our findings, a substantial body of literature suggests that elevated depressive symptoms and clinical depression are associated with poorer cognitive ability, dementia, and Alzheimer’s disease [13–16]. Our findings also concur with most [25,26] but not all earlier findings [27] suggesting that elevated depressive symptoms on their own do not accelerate cognitive decline in older people. Finally, we also found that diabetes is inversely associated with cognitive ability. This finding agrees with those of a review and two meta-analyses [8–10], which indicate that people with diabetes perform worse on multiple cognitive domains including memory and executive function.

Individually, neither diabetes nor depression accelerated cognitive decline with differences in both memory and executive function trajectories between participants with elevated depressive symptoms or diabetes and those free of these conditions remaining stable over time. It is noteworthy that the differences between participants with either condition and those free of both conditions remained relatively stable even when the entire sample experienced a decline in their cognitive ability i.e. the decline in memory that was experienced by all participants aged ≥65 years. Differences in the trajectories of cognitive ability between participants with elevated depressive symptoms or diabetes and those free of these conditions appear to be set earlier in life and carried over to older ages. The only category that experienced a significant worsening in their cognitive function over time (with the exception of memory among those ≥65 years) was that of participants with both diabetes and elevated depressive symptoms, which differences with the reference category got increasingly greater over time. Our findings are suggestive of a synergistic effect of diabetes and depression on cognitive function that accelerates cognitive decline over time and significantly adds to baseline differences. This synergistic effect is not present at baseline, but develops over time and possibly accelerates cognitive decline by exacerbating pathological processes.

Numerous pathological processes and pathways might mediate the observed associations with glycaemic control, low-grade chronic inflammation, and micro- and macro-vascular diseases being particularly pertinent. Regarding hyperglycaemia, based on evidence [28], it is plausible to assume that comorbid depression in diabetes results in poorer glycaemic control, which over time leads to greater cognitive decline via chronic hyperglycaemia. Nevertheless, recent reviews do not fully support a hyperglycaemia-related explanation for the inverse association between diabetes and cognition [29,30]. Peripheral inflammation can affect central nervous system and activate central inflammatory processes in different ways [31]. The activation of central inflammatory processes might lead to alterations in brain morphology and function, which, in turn, might be related to cognitive decline [32,33]. Vascular abnormalities are also implicated in the associations between diabetes, and brain abnormalities and cognitive dysfunction [8,34], while vascular disease links depression to cognitive impairment [15]. Diabetes and depression are established cardiovascular risk factors [35,36], and their co-occurrence can result in a multitude of vascular abnormalities, which over time can affect the brain and lead to accelerated cognitive decline. Psychosocial stress, HPA axis dysregulation and elevated levels of glucocorticoids might also be part of the mechanism that leads from diabetes and depression to cognitive decline. Depression is a stress-related disorder [37] and diabetes is strongly associated with psychosocial stress [38], while stress modulates brain function and alters brain structure and likely is associated with cognitive deficits [15,37]. Further, our findings suggest that diabetes patients with comorbid depression experienced a greater cognitive decline over time partially because they engaged in unhealthy behaviours such as physical inactivity. Previous findings suggest that unhealthy behaviours do not explain the associations between depression and cognitive decline [20] or dementia [18], but these findings come from studies of diabetes patients, which are expected to be different in terms of unhealthy behaviours compared with the general population. Socioeconomic position is also very relevant to the examined associations and explained a considerable part of them. The important role of socioeconomic position in the observed associations highlights their life course dimension and suggests that they can partially be a result of experiences of social disadvantage and lower socioeconomic position at earlier stages of the life course.

The use of a large national sample of community-dwellers aged ≥50 years makes our findings more applicable to the general population of older people. The long follow-up of eight to ten years and the use of repeated measurements allowed a thorough examination of the effect of diabetes and elevated depressive symptoms on different domains of cognitive function. The age stratification of our analyses led to an identification of significant age differences in the examined associations, while the use of imputed data is expected to minimise the effect of post-baseline non-response on our findings and reduce attrition bias [39].

Despite these advantages, our study has several weaknesses that need to be acknowledged. ELSA achieved a baseline response rate of 70%, which is good, but nevertheless leaves space for non-response bias to influence our findings. Further, the exclusion of older people who were not community-dwellers at baseline is expected to reduce to an extent the applicability of our findings to institutionalised older people. The use of self-reported doctor-diagnosed diabetes, despite the partial validation of these data against diabetes medication data, might have led to a misclassification of diabetes cases and bias to a minor extent our findings. The lack of data on concomitant medication and history of depression and inability to adjust our models for these factors is an additional limitation that might have influenced to some extent our findings. The use of the shortened 8-item CES-D, which focuses on a limited number of depressive symptoms that were experienced by the respondents during the week preceding their ELSA interview and which statistical invariance between participants with and without diabetes has not been established, is an additional limitation. The lack of baseline BMI data and subsequent use of BMI data that predated the baseline interview is a minor limitation of our work, which given the chronological proximity of the employed BMI data to the baseline is unlikely to influence our findings in a meaningful way. Another minor limitation that pertains to the non-experimental nature of our study is that the time interval between the immediate and delayed recall tests is not fixed and fluctuations in this time interval might have affected participants’ performance in the delayed recall test. Nevertheless, given the design of the ELSA cognitive interview and the strict order of the cognitive tests, we are confident that fluctuations in the time that elapsed between the two recall tests have not affected our findings in any noticeable way. Finally, the use of a sample of community-dwellers precluded us from examining the role of diabetes duration, severity and management in the observed associations.

The long-term effects of diabetes and elevated depressive symptoms on memory and executive function are significant, but rather small. Individually, neither elevated depressive symptoms nor diabetes accelerate cognitive decline over time. Conversely, the co-occurrence of diabetes and elevated depressive symptoms accelerates cognitive decline, especially among those aged 50–64 years. Future research should concentrate on explaining the biological mechanisms of the synergistic effect of comorbid depression and diabetes on cognition and identifying therapeutic targets. There is also need to examine whether diabetes mediates the association between early-onset depression and cognitive decline. Identifying and treating people with comorbid diabetes and depression, especially those at the beginning of midlife, should be a priority in clinical practice.

## Supporting information

S1 File Table A in S1 File. The prospective association between type 2 diabetes, elevated depressive symptoms and word recall summary score (memory) over 10 years in 10,524 participants aged ≥50 years. Table B in S1 File. The prospective association between type 2 diabetes, elevated depressive symptoms and word recall summary score (memory) over 10 years in 5,133 participants aged ≥50 years. Table C in S1 File. The prospective association between type 2 diabetes, elevated depressive symptoms and animal naming score (executive function) over 8 years in 10,524 participants aged ≥50 years. Table D in S1 File. The prospective association between type 2 diabetes, elevated depressive symptoms and animal naming score (executive function) over 8 years in 5,641 participants aged ≥50 years.
(DOCX)
