# Supplementary material for: Type 2 diabetes, depressive symptoms and trajectories of cognitive decline in a national sample of community-dwellers: A prospective cohort study
Source: PLoS One. 2017 Apr 17;12(4):e0175827. doi: 10.1371/journal.pone.0175827 (PMC5393617; doi:10.1371/journal.pone.0175827)
Supplement: S1 File — Table A in S1 File. The prospective association between type 2 diabetes, elevated depressive symptoms and word recall summary score (memory) over 10 years in 10,524 participants aged ≥50 years. Table B in S1 File. The prospective association between type 2 diabetes, elevated depressive symptoms and word recall summary score (memory) over 10 years in 5,133 participants aged ≥50 years. Table C in S1 File. The prospective association between type 2 diabetes, elevated depressive symptoms and animal naming score (executive function) over 8 years in 10,524 participants aged ≥50 years. Table D in S1 File. The prospective association between type 2 diabetes, elevated depressive symptoms and animal naming score (executive function) over 8 years in 5,641 participants aged ≥50 years. (DOCX) [file pone.0175827.s001.docx]

**Online Supplementary Material**

Table A presents the analysis of the associations between type 2 diabetes, elevated depressive symptoms and word recall (memory) summary score over 10 years in 10,524 participants aged ≥50 years. The models presented in S1 Table are identical to those of the main analysis (see Table 2), but for their estimation we exclusively used the observed data. No imputed data were used in the estimation of the models presented in S1 Table.

Table B presents the analysis of the associations between type 2 diabetes, elevated depressive symptoms and word recall (memory) summary score over 10 years in 5,133 participants aged ≥50 years. The models presented in S2 Table are identical to those presented in S1 Table, but in addition the sample excludes all participants who missed the last interview that the memory score was measured, that is the fifth follow-up ELSA interview in 2012-13.

Table C presents the analysis of the associations between type 2 diabetes, elevated depressive symptoms and animal naming (verbal fluency/executive function) score over 8 years in 10,524 participants aged ≥50 years. The models presented in S3 Table are identical to those of the main analysis (see Table 3), but for their estimation we exclusively used the observed data. No imputed data were used in the estimation of the models presented in S3 Table.

Table D presents the analysis of the associations between type 2 diabetes, elevated depressive symptoms and animal naming (verbal fluency/executive function) over 8 years in 5,641 participants aged ≥50 years. The models presented in S4 Table are identical to those presented in S3 Table, but in addition the sample excludes all participants who missed the last interview that the animal naming score was measured, that is the fourth follow-up ELSA interview in 2010-11.

**Table A. The prospective association between type 2 diabetes, elevated depressive symptoms and word recall summary score (memory) over 10 years in 10,524 participants aged ≥50 years**

|  | **Without diabetes and elevated depressive symptoms** | **With diabetes, but without elevated depressive** | **Without diabetes, but with elevated depressive symptoms** | **With both diabetes and elevated depressive symptoms** |
| --- | --- | --- | --- | --- |
|  | **Participants aged 50 to 64 (n=5512)** | | | |
| **No. of participants** | 4493 | 187 | 761 | 71 |
| **Slope (rate of decline)^a^** |  |  |  |  |
| **Model 4^b^** | 1.00 (reference) | -0.65 (-0.99 to -0.32)^***^ | -0.62 (-0.80 to -0.44)^***^ | -1.23 (-1.78 to -0.67)^***^ |
| **Model 5^c^** | 1.00 (reference) | -0.46 (-0.90 to -0.02)^*^ | -0.64 (-0.87 to -0.40)^***^ | -0.35 (-1.07 to 0.37) |
| **Slope acceleration (exposure*time interaction)^a^** |  |  |  |  |
| **Model 5** | 1.00 (reference) | -0.08 ( -0.18 to 0.03) | 0.01 (-0.05 to 0.06) | -0.36 (-0.56 to -0.17)^***^ |
|  | **Participants aged ≥65 years (n=5012)** | | | |
| **No. of participants** | 3782 | 367 | 765 | 98 |
| **Slope (rate of decline)^a^** |  |  |  |  |
| **Model 4^b^** | 1.00 (reference) | -0.38 (-0.66 to -0.10)^**^ | -0.52 (-0.73 to -0.31)^***^ | -0.63 (-1.17 to -0.10)^*^ |
| **Model 5^c^** | 1.00 (reference) | -0.37 (-0.73 to -0.01)^*^ | -0.37 (-0.64 to -0.10)^**^ | -0.71 (-1.41 to -0.02)^*^ |
| **Slope acceleration (exposure*time interaction)^a^** |  |  |  |  |
| **Model 5^c^** | 1.00 (reference) | -0.01 ( -0.11 to 0.10) | -0.07 (-0.15 to 0.01) | 0.04 (-0.18 to 0.26) |

^a^ The estimates are β regression coefficient (95% confidence intervals)

^b^ Model 4 is adjusted for age, sex, marital status, self-reported chronic conditions i.e. heart disease, stroke, hypertension and chronic lung disease, education, occupational class, physical activity, smoking alcohol, consumption, and body mass index (including a category for missing BMI values)

^c^ Model 5 is in addition adjusted for the exposure*time interaction term

*** p value≤0.001

** p value≤0.01

* p value≤0.05

**Table B. The prospective association between type 2 diabetes, elevated depressive symptoms and word recall summary score (memory) over 10 years in 5,133 participants aged ≥50 years**

|  | **Without diabetes and elevated depressive symptoms** | **With diabetes, but without elevated depressive** | **Without diabetes, but with elevated depressive symptoms** | **With both diabetes and elevated depressive symptoms** |
| --- | --- | --- | --- | --- |
|  | **Participants aged 50 to 64 (n=3282)** | | | |
| **No. of participants** | 2735 | 106 | 413 | 28 |
| **Slope (rate of decline)^a^** |  |  |  |  |
| **Model 4^b^** | 1.00 (reference) | -0.58 (-0.99 to -0.17)^**^ | -0.53 (-0.75 to -0.31)^***^ | -1.22 (-1.99 to -0.44)^**^ |
| **Model 5^c^** | 1.00 (reference) | -0.41 (-0.95 to 0.13) | -0.57 (-0.86 to -0.27)^***^ | 0.10 (-0.93 to 1.12) |
| **Slope acceleration (exposure*time interaction)^a^** |  |  |  |  |
| **Model 5^c^** | 1.00 (reference) | -0.05 ( -0.17 to 0.06) | 0.01 (-0.05 to 0.07) | -0.43 (-0.64 to -0.21)^***^ |
|  | **Participants aged ≥65 years (n=1851)** | | | |
| **No. of participants** | 1518 | 109 | 203 | 21 |
| **Slope (rate of decline)^a^** |  |  |  |  |
| **Model 4^b^** | 1.00 (reference) | -0.46 (-0.91 to -0.02)^*^ | -0.32 (-0.66 to 0.01) | -0.61 (-1.59 to 0.36) |
| **Model 5^c^** | 1.00 (reference) | -0.31 (-0.89 to 0.26) | -0.13 (-0.56 to 0.31) | -0.54 (-1.81 to 0.75) |
| **Slope acceleration (exposure*time interaction)^a^** |  |  |  |  |
| **Model 5^c^** | 1.00 (reference) | -0.05 ( -0.18 to 0.08) | -0.07 (-0.17 to 0.03) | -0.03 (-0.31 to 0.26) |

^a^ The estimates are β regression coefficient (95% confidence intervals)

^b^ Model 4 is adjusted for age, sex, marital status, self-reported chronic conditions i.e. heart disease, stroke, hypertension and chronic lung disease, education, occupational class, physical activity, smoking alcohol, consumption, and body mass index (including a category for missing BMI values)

^c^ Model 5 is in addition adjusted for the exposure*time interaction term

*** p value≤0.001

** p value≤0.01

* p value≤0.05

**Table C. The prospective association between type 2 diabetes, elevated depressive symptoms and animal naming score (executive function) over 8 years in 10,524 participants aged ≥50 years**

|  | **Without diabetes and elevated depressive symptoms** | **With diabetes, but without elevated depressive** | **Without diabetes, but with elevated depressive symptoms** | **With both diabetes and elevated depressive symptoms** |
| --- | --- | --- | --- | --- |
|  | **Participants aged 50 to 64 (n=5512)** | | | |
| **No. of participants** | 4493 | 187 | 761 | 71 |
| **Slope (rate of decline)^a^** |  |  |  |  |
| **Model 4^b^** | 1.00 (reference) | -0.29 (-1.03 to 0.45) | -1.02 (-1.41 to -0.63)^***^ | -1.47 (-2.66 to -0.28)^**^ |
| **Model 5^c^** | 1.00 (reference) | -0.10 (-1.03 to 0.84) | -0.96 (-1.46 to -0.46)^***^ | -0.39 (-1.92 to 1.13) |
| **Slope acceleration (exposure*time interaction)^a^** |  |  |  |  |
| **Model 5^c^** | 1.00 (reference) | -0.09 ( -0.34 to 0.17) | -0.03 (-0.16 to 0.11) | -0.52 (-0.97 to -0.06)* |
|  | **Participants aged ≥65 years (n=5012)** | | | |
| **No. of participants** | 3782 | 367 | 765 | 98 |
| **Slope (rate of decline)^a^** |  |  |  |  |
| **Model 4^b^** | 1.00 (reference) | -0.57 (-1.08 to -0.07)^*^ | -0.85 (-1.23 to -0.47)^***^ | -1.44 (-2.40 to -0.49)^**^ |
| **Model 5^c^** | 1.00 (reference) | -0.31 (-0.98 to 0.36) | -0.58 (-1.08 to -0.09)^*^ | -0.12 (-1.40 to 1.16) |
| **Slope acceleration (exposure*time interaction)^a^** |  |  |  |  |
| **Model 5^c^** | 1.00 (reference) | -0.13 ( -0.35 to 0.09) | -0.14 (-0.31 to 0.03) | -0.70 (-1.14 to -0.25)^**^ |

^a^ The estimates are β regression coefficient (95% confidence intervals)

^b^ Model 4 is adjusted for age, sex, marital status, self-reported chronic conditions i.e. heart disease, stroke, hypertension and chronic lung disease, education, occupational class, physical activity, smoking alcohol, consumption, and body mass index (including a category for missing BMI values)

^c^ Model 5 is in addition adjusted for the exposure*time interaction term

*** p value≤0.001

** p value≤0.01

* p value≤0.05

**Table D. The prospective association between type 2 diabetes, elevated depressive symptoms and animal naming score (executive function) over 8 years in 5,641 participants aged ≥50 years**

|  | **Without diabetes and elevated depressive symptoms** | **With diabetes, but without elevated depressive** | **Without diabetes, but with elevated depressive symptoms** | **With both diabetes and elevated depressive symptoms** |
| --- | --- | --- | --- | --- |
|  | **Participants aged 50 to 64 (n=3456)** | | | |
| **No. of participants** | 2858 | 114 | 452 | 32 |
| **Slope (rate of decline)^a^** |  |  |  |  |
| **Model 4^b^** | 1.00 (reference) | 0.16 (-0.73 to 1.06) | -0.99 (-1.48 to -0.51)^***^ | -0.91 (-2.57 to 0.74) |
| **Model 5^c^** | 1.00 (reference) | 0.62 (-0.54 to 1.77) | -0.88 (-1.51 to -0.26)^**^ | 0.56 (-1.58 to 2.70) |
| **Slope acceleration (exposure*time interaction)^a^** |  |  |  |  |
| **Model 5^c^** | 1.00 (reference) | -0.17 ( -0.45 to 0.10) | -0.04 (-0.19 to 0.10) | -0.56 (-1.07 to -0.05)^*^ |
|  | **Participants aged ≥65 years (n=2185)** | | | |
| **No. of participants** | 1767 | 139 | 251 | 28 |
| **Slope (rate of decline)^a^** |  |  |  |  |
| **Model 4^b^** | 1.00 (reference) | -0.47 (-1.24 to 0.30) | -0.55 (-1.15 to 0.04) | -1.33 (-2.97 to 0.32) |
| **Model 5^c^** | 1.00 (reference) | -0.21 (-1.22 to 0.80) | -0.21 (-0.98 to 0.57) | 0.72 (-1.46 to 2.90) |
| **Slope acceleration (exposure*time interaction)^a^** |  |  |  |  |
| **Model 5^c^** | 1.00 (reference) | -0.10 ( -0.36 to 0.15) | -0.14 (-0.33 to 0.06) | -0.79 (-1.34 to -0.24)^**^ |

^a^ The estimates are β regression coefficient (95% confidence intervals)

^b^ Model 4 is adjusted for age, sex, marital status, self-reported chronic conditions i.e. heart disease, stroke, hypertension and chronic lung disease, education, occupational class, physical activity, smoking alcohol, consumption, and body mass index (including a category for missing BMI values)

^c^ Model 5 is in addition adjusted for the exposure*time interaction term

*** p value≤0.001

** p value≤0.01

* p value≤0.05
